# Indian vaccine innovation: the case of Shantha Biotechnics

**DOI:** 10.1186/1744-8603-7-9

**Published:** 2011-04-20

**Authors:** Justin Chakma, Hassan Masum, Kumar Perampaladas, Jennifer Heys, Peter A Singer

**Affiliations:** 1 McLaughlin-Rotman Centre for Global Health, University Health Network and University of Toronto, 101 College Street Suite 406, Toronto ON, M5G 1L7 Canada

## Abstract

**Background:**

Although the World Health Organization had recommended that every child be vaccinated for Hepatitis B by the early 1980s, large multinational pharmaceutical companies held monopolies on the recombinant Hepatitis B vaccine. At a price as high as USD$23 a dose, most Indians families could not afford vaccination. Shantha Biotechnics, a pioneering Indian biotechnology company founded in 1993, saw an unmet need domestically, and developed novel processes for manufacturing Hepatitis B vaccine to reduce prices to less than $1/dose. Further expansion enabled low-cost mass vaccination globally through organizations such as UNICEF. In 2009, Shantha sold over 120 million doses of vaccines. The company was recently acquired by Sanofi-Aventis at a valuation of USD$784 million.

**Methods:**

The case study and grounded research method was used to illustrate how the globalization of healthcare R&D is enabling private sector companies such as Shantha to address access to essential medicines. Sources including interviews, literature analysis, and on-site observations were combined to conduct a robust examination of Shantha's evolution as a major provider of vaccines for global health indications.

**Results:**

Shantha's ability to become a significant global vaccine manufacturer and achieve international valuation and market success appears to have been made possible by focusing first on the local health needs of India. How Shantha achieved this balance can be understood in terms of a framework of four guiding principles. First, Shantha identified a therapeutic area (Hepatitis B) in which cost efficiencies could be achieved for reaching the poor. Second, Shantha persistently sought investments and partnerships from non-traditional and international sources including the Foreign Ministry of Oman and Pfizer. Third, Shantha focused on innovation and quality - investing in innovation from the outset yielded the crucial process innovation that allowed Shantha to make an affordable vaccine. Fourth, Shantha constructed its own cGMP facility, which established credibility for vaccine prequalification by the World Health Organization and generated interest from large pharmaceutical companies in its contract research services. These two sources of revenue allowed Shantha to continue to invest in health innovation relevant to the developing world.

**Conclusions:**

The Shantha case study underscores the important role the private sector can play in global health and access to medicines. Home-grown companies in the developing world are becoming a source of low-cost, locally relevant healthcare R&D for therapeutics such as vaccines. Such companies may be compelled by market forces to focus on products relevant to diseases endemic in their country. Sanofi-Aventis' acquisition of Shantha reveals that even large pharmaceutical companies based in the developed world have recognized the importance of meeting the health needs of the developing world. Collectively, these processes suggest an ability to tap into private sector investments for global health innovation, and illustrate the globalization of healthcare R&D to the developing world.

## Background

For many years, Western investors were attracted by the prospect of outsized returns in the biotechnology industry. Amgen's initial investors received returns of almost 100 fold after only 3 years [1], while Genentech's patents generated hundreds of millions of dollar in royalties [2].

Even as early enthusiasm for biotechnology's potential commercial returns cooled in the West over the past decade [3], the globalization of biotechnology spurred the creation of a range of biotechnology companies in emerging markets. This select sub-set of the developing world including China, India, Brazil and South Africa experienced marked economic development over the last two decades, and now has significant scientific and financial capital under the stewardship of relatively stable political systems.

In India, an industry-led biopharma sector emerged, with large companies like Ranbaxy in the 1970s and 1980s leveraging recognition of process rather than product patents from the Patent Act of 1970 to rapidly expand and become internationally known for manufacturing generic drugs [4,5]. However, India also has dozens of lesser known small to mid size innovative biotechnology companies, most of which have developed since 1990 [6,7]. These companies have grown to play a pivotal role in ensuring access to medicines globally by serving as a low-cost source of global health innovation, particularly in the vaccine arena.

In this article, we describe and analyze the history and lessons of one of these vaccine innovators, Shantha Biotechnics. In late 2009, in a landmark deal for the Indian biotech industry, Shantha was acquired by the multinational giant Sanofi-Aventis (France) at a valuation of USD$784 million [8]. Founded by Dr. K.I. Varaprasad Reddy in 1993, Shantha was one of the first Indian biotechs to create a recombinant product, obtaining World Health Organization (WHO) prequalification for its Hepatitis B vaccine in 2002 [7]. Since then, the firm has grown to 750 employees and brought 11 novel products to market. In 2009, Shantha sold over 120 million doses of Hepatitis B vaccine to dozens of developing countries around the world, had revenues exceeding USD$90 million [9] and maintained a sophisticated pipeline of biologics including human monoclonal antibodies.

We begin by describing the history of Shantha's unique evolution from a start-up to a significant vaccine player, noting key challenges and decision points in the process with respect to financials, corporate strategy and health impact. We then analyze the Shantha case as a whole as an illustration of the globalization of healthcare R&D, to draw out key lessons for scientists, entrepreneurs, and policy-makers. The goal of this description and analysis is to accurately describe the case, and suggest observations and lessons that may be applicable to those who wish to start, partner with, or invest in R&D-intensive private-sector firms in emerging markets that tackle global health challenges.

## Methods

This analysis is based on multiple site visits and face to face interviews with key members of the Shantha Biotechnics team over the past five years, as well as analysis of secondary material. Where not specifically noted or referenced, the report is based on these interviews.

We chose a combination of the case study and grounded research methods as the most appropriate ones to examine complex phenomena in context [10]. Shantha Biotech was chosen on the basis of being identified by previous studies as one of the first innovative Indian biotechs, and one of the few to be acquired by a large foreign pharmaceutical multinational company [8]. It was part of a set of fourteen case-studies focused on healthcare product development and investment occurring in India and Africa (see e.g. [11]). This particular case-study also builds on other case-studies of biotechnology innovation in emerging markets that our research group has published [6]. We have published a significantly abbreviated version of the case-study in another journal [12]

Interviewees were selected based on purposive sampling. We analyzed transcripts from semi-structured, face-to-face interviews that took place in Hyderabad with a total of 16 Shantha representatives on four separate occasions (January 2005, 2006, March 2008, October 2008). Interviewees included Dr. K.I. Varaprasad, Shantha's CFO N. Rajasekar and CSO Ashok Khar as well as Georges Hibon (a Director of BioMerieux Alliance). These were followed by email follow-ups with Shantha and BioMerieux executives in August-October 2009.

We also analyzed background documents on the Indian biotechnology industry from the peer-reviewed literature and news reports; books published by biotechnology industry and innovation academics; Indian and USPTO patents filed by Shantha, reports and presentations from the Government of India, World Health Organization, and World Intellectual Property Organization; institutional websites such as PATH, NIH, International Vaccine Initiative; and firm websites of Shantha, Sanofi-Aventis and BioMerieux. The firm was asked to fact-check the data derived from our analysis prior to submission to ensure it was up-to-date and accurate. Analysis of transcripts was supported by qualitative data analysis software ATLASti and NVivo. This study was approved by the Office of Research Ethics of the University of Toronto.

## Results

### The Hepatitis B Tragedy

Over 100,000 Indians die every year from Hepatitis B infection [13]. About 40 million individuals are chronically infected, and 4% of the Indian population are carriers. As a serious liver infection, it is transmitted through exposure to infectious blood or bodily fluids, including during childbirth. By the early 1980s, the WHO recommended that every child be vaccinated for Hepatitis B, but inexpensive recombinant vaccines had not been developed. Merck and GlaxoSmithKline (formerly SmithKlineBeecham) had developed recombinant vaccines in 1986, and they held a monopoly with over 90 other patents covering manufacturing processes such as isolation and purification [14]. In the late 1980s, prices were as high as USD$23 a dose. Plasma-derived vaccines had been produced in India since 1981, but concerns arose around the capacity to produce large quantities of plasma-derived vaccines, and about their safety since they are derived from human blood [14]. With most Indian families living on USD $1 a day with multiple children and three doses required per child, vaccination was simply unaffordable [15].

### An Engineer with a Cause

Dr. K.I. Varaprasad Reddy reported that he discovered the extent of the issue when he attended a WHO conference in 1992, and learned that what was needed was an inexpensive generic biotech vaccine. He felt that the vaccine would have to be produced in-country rather than imported. The Indian biotech industry at that time was focused on generic pharmaceutical products, and was not yet involved in innovative biotechnology [4]. Recombinant technology did not exist within the country [6]. When Dr. Varaprasad approached a Western firm for a technology transfer he was told that, essentially, "India cannot afford such high technology vaccines. India does not require vaccines. And even if you can afford to buy the technology, your scientists cannot understand recombinant technology in the least." Despite being trained as an electrical engineer with no biotech R&D experience and just an MBA, Dr. Varaprasad was motivated by this challenge and felt that the science was something he could delegate to an experienced team of scientists.

### Building the Dream

With an idea in mind and strong convictions, Dr. Varaprasad began to seek capital for this new venture. Although he visited every major Indian bank, they were unwilling to fund early-stage start-ups with no revenue, and had little understanding of the biotech industry at large. But Varaprasad persisted, and raised $1.2 M USD by selling his father's properties, and seeking investment from family and friends. As Dr. Varaprasad himself had no experience in biological research, he contacted hundreds of expatriate Indian scientists, two of whom he persuaded to join him. Shantha was founded in 1993 with few resources, but much hope. As one of the scientists, M.K. Sudhir, stated: "If you ask me if I would go through it again, I would have to think twice. At that point, it was a missionary zeal. There was no precursor for this kind of product in India."

Shantha incubated inside Osmania University at Hyderabad, but the company was relocated because of perceived institutional politics. By 1995, Shantha had exhausted its initial investment and was on the verge of bankruptcy. Dr. Varaprasad fortunately found an unexpected ally in the Foreign Minister of the Sultanate of Oman, H. E. Yusuf Bin Alawi Abdullah, who wanted an affordable vaccine for his own citizenry. Oman injected $1.2 million USD in equity for a 50% stake in the firm, which allowed Shantha to move into a new facility at the Centre for Cellular and Molecular Biology in Hyderabad. This investment carried them forward to 1997 [See Table 1].

**Table 1:** Shantha Timeline

Year	Milestone
1992	Dr. Varaprasad attends immunization conference in Geneva; Hep-B idea forms

1993	Shantha Biotechnics is born, staff works out of Osmania University

1994	Shantha Biotechnics moves to Centre for Cellular and Molecular Biology

1995	Oman invests $1.2 million in equity; Shantha moves into own facility

1997	Shantha's Hepatitis B vaccine, Shanvac-B, launched (first recombinant health product in India)

1998	Shantha sells 22 million doses of Shanvac-B this year, far exceeding expectations

1999	Comparative study proving the high quality of Shanvac-B published in *Vaccine*

2000	Morgan Stanley and State Bank of Indian Mutual Fund invest $10 million in equity

2002	Shantha introduces first bio-therapeutic product, Interferon α 2b, onto the market Shantha receives WHO pre-qualification for Shanvac-B

2005	Shantha introduces first combination vaccine onto market - Shantetra (diphtheria, pertussis, tetanus, hepatitis B)

2007	Merieux Alliance picks up 60% stake in Shantha, which it later expands to 80%

2009	Shantha wins a $340 M USD contract from UNICEF for pentavalent vaccines
	Sanofi-Aventis acquires an 80% controlling stake valuing the firm at $784 M USD

### A Process Innovation

After four years of supporting his scientists, Dr. Varaprasad's patience paid off. In 1997, Shanvac-B, India's first home-grown recombinant product, launched at a price of about USD $1 a dose. The vaccine was produced in *Pichia pastoris*, a yeast system different from that used by the original inventors of the vaccine. Although at the time *Pichia pastoris *was being used for research purposes, Dr. Varaprasad was told by the manufacturers of the expression system that Shantha was the first company to use *Pichia pastoris *to produce a commercial product [16]. Its Shanvac-B process innovation earned them two process patents, a beneficiary of India's preferential treatment of process patents over product patents in its Patent Act of 1970 [17]. According to Dr. K. V. Sudhir, one of the pioneering scientists, "in hindsight... [it] was a major factor in us being so successful" because it led to better yields and more efficient purification compared to even multinational processes [16].

### From Lab to Village

According to the annual reports of Shantha, analysts projected first year sales of only $100 000 USD given Shanvac-B's low price, but actual sales in 1997 exceeded $1.6 million USD. Shanvac-B was launched at around USD $1 per dose because, in Dr. Varaprasad's words "...my gut feeling [was], unless it is made for one dollar, nobody can afford this." But it was not a charitable act - net profit margins were reported to be around 20% [18].

Initial sales were financed almost entirely by the private-sector as the public health agencies in India did not have a mandated vaccine schedule yet, and most healthcare services were (and continue to be) provided by private healthcare. The price was a fraction of that charged by the competition [19], based on Dr. Varaprasad's desire to make the vaccine affordable to Indian citizens rather than charging what the market would bear. Consumption of vaccine increased from a few hundred thousand doses in early 1990s to over 30 million doses in November 2008 with increasing involvement of donor and public health agencies [20]. Prices in the Indian market reportedly dropped from about $15 to as low as $0.23 USD [6,14].

Revenues exceeding $90 million USD in 2009 have validated Shantha's high-volume, low-margin strategy [21]. This rapid success was partly due to mentorship from a large multinational pharmaceutical for developing good manufacturing practices and regulatory acumen. Pfizer (New York, NY) was impressed enough with the quality that it agreed to co-market Shantha's Hepatitis B vaccine under the HepaShield brand in India in 2002 [6]. In 2000, Morgan Stanley and the State Bank of India Mutual Fund invested USD $10 million in a private round of equity raising to build manufacturing facilities.

### A Tradition of Innovation

Shantha continued to employ process innovations for subsequent products. Its second product, interferon alpha 2-b (Shanferon), was also produced in *Pichia pastoris *reportedly marking the first time this molecule was produced commercially in yeast rather than the traditional bacterial system [22]. Shanferon was reportedly priced at Rs 300 ($USD 6.50), which was also substantially lower than the then imported price of Rs 1200 ($USD 26.00) [23]. Shantha was one of the first biotechs to produce erythropoietin in serum-free media, which quelled safety concerns regarding serum use in manufacturing [24]. In using these new processes, Shantha's scientists not only had to alter the method by which they produced their product, but also the entire purification process. Although it took additional time to develop good manufacturing practices that adhered to ICH-WHO norms, the decision to focus on process innovation right from the beginning led Shantha to become the first Indian company to be prequalified by the WHO [6]. The initial investment in quality control helped accelerate approval for its later vaccines: Shantha now has four vaccines that are WHO pre-qualified [25]

After Hepatitis B, Shantha started development programs for interferon alpha 2-b, vaccines for rotavirus, HPV, pneumococcal viruses and oral cholera, and set up a subsidiary in the United States to develop monoclonal antibodies for cancer indications [26,27]. This was only made possible by Shantha's commitment to invest 12-25% of its profits back into R&D every year [28] - a number higher than its typical Indian compatriots, and an ambitious goal to keep new products coming onto the market every one or two years. "The criterion was simply to look at products that were relevant to India and the other developing country's needs," said Shantha's CSO Ashok Khar.

Shantha facilitated this pipeline expansion through not only home-grown efforts, but also partnerships with the US National Institutes of Health, Bill & Melinda Gates Foundation, John Hopkins University, and PATH [29]. By building a close relationship with the Center for Cellular and Molecular Biology (CCMB) and other Indian research institutes [30], Shantha has also benefited from access to local scientists and R&D ideas for novel expression vectors. For example, it worked with the International Center for Genetic Engineering in Biotechnology in New Delhi, India for novel kinase inhibitors for cancer with the potential for revenue sharing.

Its focus on innovation and quality to meet WHO prequalification standards led to a higher cost structure than its domestic competitors who did not meet these standards. In recent years, this has limited domestic sales. However, Dr. Varaprasad believes Shantha is able to compensate through greater access to international markets. This focus on innovation and quality was demonstrated when a multinational competitor ran a campaign that questioned the quality of its Hepatitis B vaccine [31]. A double-blind comparative study showed that Shanvac-B was equivalent or superior to the competitor's product on all counts - immunogenicity was found to be higher, side effects fewer, and seroconversion was high enough that only two doses of the vaccine were required in contrast to the three doses required by the competition [32].

### Joined, but Not Beaten

The success of Shanvac-B was arguably an important step for the country's health technology sector. It provided a proof of concept that scientists working in India were able to conduct advanced biotech R&D. Since then, several companies have followed in its footsteps. There are now five Indian companies that produce the Hepatitis B vaccine [33], and as noted by Shantha's Executive Director Mr. Khalil Ahmed, "Everybody and their cousin have started a biotech company in India." The Indian biotech sector, which was almost non-existent in the early 1990s, is on track to generate at least $7 billion in annual revenues by the end of 2010 [34,35].

Marketing proved critical to maintaining competitiveness. Shantha has a sales force of 175 people that market drugs directly to doctors using conferences and seminars to reduce mark-ups through distributors that were said to reach up to 200% by the time the product reaches the public. Although Shantha conducted vaccination and education camps to increase public awareness with regards to the importance of being vaccinated, Shantha's Executive Director Khalil Ahmed observed that Indian doctors felt that this is the "dirtiest thing companies could do, by selling cheaply to end-users." Given the respect that doctors command in India, Shantha executives felt having their support was critical. Marketing to physicians allowed Shantha to distinguish itself from the counterfeits and justify its premium. This was reflected by Pfizer's decision to purchase and sell Shanvac-B as a branded generic by leveraging doubts and concerns among Indians about counterfeit or low-quality drugs [6,36].

As Shantha's reputation has grown, several emerging markets have engaged Shantha in R&D and clinical collaborations for recombinant vaccines. The International Vaccine Institute (South Korea) asked Shantha for help conducting clinical trials in Kolkata and co-developing its own new-generation oral cholera vaccine [37]. Despite recommendations from the WHO for use of these new vaccines in 2001, only Vietnam was locally producing oral cholera vaccines [38]. However, an analysis of this vaccine showed that for it to comply with WHO guidelines, the vaccine needed to be reformulated and its production technology modified. The one internationally licensed cholera vaccine, Dukoral produced by Crucell/SBL Vaccines, was too expensive at $18/shot in India. Following successful reformulation in early 2009, Shantha was selected by IVI to manufacture this new vaccine, and the price has since reportedly dropped to $2/shot [39]. Similarly, Shantha has partnered with Pediatric Dengue Vaccine Initiative (South Korea) to run a Phase I clinical trial for its dengue vaccine [40].

### The French Attraction

Shantha's success led to international attention in 2006 when Merieux-Alliance (France) acquired a 60% stake in Shantha after the Omani investors sought an exit [41]. However, Dr. Varaprasad stated that he had no intention of ending up as a "glorified employee of a multinational company." Shantha insisted on maintaining its focus on providing affordable vaccines to the poor, and being able to retain its Indian characteristics such as the company name, management, and philosophies. The transition led to Shantha sharpening its focus on vaccines. Its monoclonal antibody development program wound down, and Shantha moved away from performing contract research services. Dr. Varaprasad warns fellow entrepreneurs in emerging markets to be aware of these challenges in striking a balance between health impact and firm value. He admitted that the transition led him to think about retiring, although he does concede that Merieux is focused on the welfare of Indians. "They don't want to take risks like an entrepreneur does."

The acquisition helped Shantha to further build its reputation internationally and open new markets. Almost 60% of Shantha's revenues came from exports at the time because the Indian Government had not added the Hepatitis B vaccine to its national immunization schedule. In 2009, the firm was awarded a USD$340 million UNICEF contract for pentavalent vaccines through 2010-2012, and in parallel, India adopted the vaccines for its immunization schedule at the recommendation of the WHO [42]. This access to international markets proved useful again in 2009 when rumors emerged that GlaxoSmithKline and other multinationals were interested in bidding on Shantha [43]. These rumors culminated in an announcement on July 27th, 2009 that Sanofi-Aventis had acquired a controlling stake in Shantha at a valuation of $784 million USD.

## Discussion

Our analysis based on interviews and the existing body of published secondary literature finds that Shantha's commitment to local health problems helped it to achieve its financial success. The late C.K. Prahalad identified the existence of a significant market among the lower-income populations of the world - the so-called "bottom of the pyramid" [44,45]. While the market size is certainly large, the process of actually reaching these customers is difficult for large firms, let alone a small start-up with serious short-term financial concerns. It is a delicate balancing act between developing affordable health solutions for the poor and increasing firm valuation. While Shantha managed to achieve this balance-having provided affordable vaccines both domestically and internationally, while still being financially successful-questions remain regarding the degree to which it can continue to do so under foreign ownership, and the road to be followed by other biotechs in emerging markets with strong economic growth and stable political environments that wish to emulate its successful balance.

As one of the first innovative biotechs in emerging markets to be acquired for a significant valuation ($768 million USD) by a major pharmaceutical multinational, it remains uncertain whether Shantha will be able to maintain its low prices and commitment to the local health markets under foreign ownership. We outline several lessons for how these biotech innovators in poorer but rapidly developing countries such as India (whom we term Southern innovators in light of their traditional geographic location) might successfully achieve this balance between local health impact and financial returns.

First, Southern innovators should identify a therapeutic area where cost efficiencies can be achieved for reaching the base of the pyramid, and combine this with strong leadership skills [44,45]. The idea that the poor are a sustainable and ideal initial market has been a common thread in previous studies describing successful Southern innovators, especially vaccine manufacturers including Indian Immunologicals and the Serum Institute [4,6,11].

Dr. Varaprasad recognized that expensive multinational recombinant vaccines had minimal market penetration, and had not tapped into the full potential of the vaccine. He realized that he could leverage India's homegrown scientists, the lower cost of labor, process innovation, and a low-margins business strategy to exploit this opportunity. Executing this insight ultimately depended on Dr. Varaprasad's strong management skills, and reflects why venture capitalists often emphasize the importance of the management team [46]. In spite of his electrical engineering background, Dr. Varaprasad was able to build a successful biotech. In fact, it may have been because Dr. Varaprasad was outside the mainstream that he was able to attempt something truly bold; he said: "A lot of scientists need reality checks... science is one part of the whole thing." Ultimately, his commitment to the local health needs, ability to build a strong R&D organization, and vision were key ingredients to Shantha's success.

Second, Southern innovators should persistently seek investments and partnerships from nontraditional and international sources. Domestic early-stage financing still remains scarce even fifteen years after Dr. Varaprasad created Shantha Biotech [6], especially given India's current stage of economic development, where manufacturing and service-oriented businesses have significant potential for high return on investment with relatively lower risk compared to pure R&D-oriented businesses. International investors from more R&D-intensive economies may be more receptive to investing in R&D-intensive start-ups than local investors, because such international investors have previously committed to and experienced such investments. This difficulty in finding financing is not entirely dissimilar to that faced by biotech innovators during the 1970s in the United States, who often had to bootstrap themselves from non-traditional angel and NIH financing (although these firms had the benefit of being preceded by semiconductor start-ups that established a critical mass of astute domestic tech investors, and liquid capital markets for such investments). Shantha was forced to grow in parallel and compete for financing with the nascent Indian IT industry during the 1990s, which offered much quicker returns.

Varaprasad was so passionate about solving the Hepatitis B challenge that he was willing to sell his father's own property. This commitment was evident to the Omani Foreign Minister and the local universities that provided free lab space. Dr. Varaprasad says that "We had a lot of sympathy from many institutions. 'These people are struggling. They want to do something on their own. No technology transfer from any country, and they want to do it on their own. Wonderful. And if they ask any help, let's do that.'"

Shantha embraced partnerships with not only research institutes such as the NIH, but also potential competitors in the form of a multinational pharmaceutical for regulatory guidance. These principles of collaboration among domestic and foreign competitors have been embraced through the founding in 2003 of the Developing Country Vaccine Manufacturers Network (DCVMN), whose members collectively supply over half of UNICEF's vaccines [47].

Third, Southern innovators should focus on innovation and quality Shantha invested in innovation from the outset, which yielded the crucial process innovation that allowed its Hepatitis B vaccine to succeed. By continuing to invest a significant proportion of its profits towards R&D [7], Shantha was able to develop a new product every one or two years - a "tick-tock" strategy similar to semiconductor manufacturer Intel's approach (Santa Clara, CA) [48]. This initial focus on process and quality innovation may have delayed Shanvac-B's launch, but it allowed Shantha to become the first Indian firm to receive WHO prequalification, and opened the door to large international contracts [See Table 2]. Obtaining this quality certification also allowed Shantha to subsidize its R&D operations through contract research work for large pharmaceutical companies. Pfizer was even willing to sell a branded generic version of Shanvac-B (HepaShield) [6].

**Table 2:** Shantha's Product Pipeline (2009)

Product Description	Development Stage	Development Partners
**Vaccines**		

Hepatitis B (Shanvac-B)	On market since 1997, post-marketing survey in progress	CCMB (India)

Japanese Encephalitis (Jencevac)	On market	Green Cross Vaccine Corporation (Korea)

Hib (ShanHib)	On market	Berna Biotech (Switzerland)

Rotavirus	Preclinical	NIH, Bill and Melinda Gates Foundation, PATH

Varicella	Preclinical	NIH

Dengue	Preclinical	Inviragen, CDC

HPV	Preclinical	NIH, NCI, John's Hopkins University

Pneumococcal	R&D	PATH

Meningococcal A (Intervax)	On market	Intervax Biologics (Canada)

Meningococcal C (Intervax)	On market	Intervax Biologics (Canada)

Cholera (oral)	Clinical trials	International Vaccine Institute, Korea

Typhoid	Clinical trials	International Vaccine Institute, Korea

**Combination Vaccines**		

MMR	R&D	-

DPT	On market	-

Shantetra: DPT, Hep B	On market	-

ShanHib-DPT: Hib, DPT	On market	-

Shan5: DPT, Hep B, Hib	On market	-

DPT, Hep B, influenza	Was expected on market 2009	-

Monoclonal Antibodies		

Lung cancer	Preclinical	-

Melanoma	Preclinical	-

Cocktail	R&D	-

**Bio-therapeutics**		

Interferon α 2b (Shanferon)	On market	-

Erythropoietin (Shanpoietin)	On market (launched 2005)	-

Streptokinase (Shankinase)	On market - discontinued	-

Insulin	On market	Biocon

GCSF	Clinical trials	-

**Diagnostics**		

Hepatitis B	On market	-

Cancer (α-feto protein)	On market	-

This focus on quality also led Shantha to recognize that for certain types of clinical trials, India's regulatory expertise was insufficient to conduct them at home [49]. For its monoclonal antibody trials for lung metastasis in melanoma, Shantha set up a San Diego-based subsidiary (Shantha West) for a reported $9 million USD in 2000 - only three years after the launch of Shanvac-B [50]. In silico development was conducted in San Diego, while wet lab work was conducted in India. Southern innovators should be realistic about capacity for home-grown manufacturing or clinical trials, and consider if it makes business sense. Approval processes in India can be slow, which has in the past resulted in uncertain regulatory processes [50]. Until recently India did not have clinical data protection as mandated by the TRIPS agreement. However, following the implementation of TRIPS, there was a significant inflow of clinical trial outsourcing to India due to its cost advantage and genetically diverse population [51,52].

Fourth, Southern innovators should realize that integrated business models are still viable in developing countries, and are arguably critical for reaching the base of the pyramid. Before its acquisition, Shantha was a fully integrated biotech that would not invest in any products for which it did not have internal capacity to execute on a significant part of the project. In the developed world, a popular business model is to become 'virtual', whereby biotechs outsource their clinical trials and even early-stage work to contract research organizations (CROs) in both mature and emerging markets [52]. Such virtual biotechs rarely develop a sales force and other downstream capabilities.

This model may not make sense for firms like Shantha, because the risks of low quality and delays in outsourcing to another domestic firm are too great. By maintaining internal development capabilities, firms are able grow from retained earnings generated by contract research work and other revenues, as Shantha did. Marketing and downstream capabilities are also critical for Southern innovators to justify the premium of their drug to potential purchasers, and to distinguish their drug from counterfeits.

While the dataset discussed in this article is limited to a single Indian vaccine firm, there are a few other Indian biotechs that are following a similar trajectory including Bharat Biotech, whose rotavirus vaccine is currently in Phase III trials, Panacea Biotech, with over half a dozen single or combination vaccines against locally relevant diseases such as cholera, encephalitis and meningitis, and Serum Institute of India, which is the world's largest producer of measles and DTP vaccines [53]. Much like how Amgen and Genentech provided a pioneering model centred on recombinant manufacturing of known biologics for over a half-dozen biotech entrants in the United States, Shantha's pioneering integrated vaccine R&D model may prove applicable for biotech firms in emerging markets over the next decade.

Further case study research on firms like Bharat Biotech, Serum Institute of India and Panacea Biotech may prove useful to deepen the lessons generated from Shantha. Although Bharat has yet to be acquired like Shantha, Bharat's reported 2007/2008 revenue was only ~ $2 million USD less than Shantha's [23]. Another limitation of the generality of our study may be Shantha's large domestic market in India, which was similarly true for China and Brazil's domestic vaccine innovators. Vaccine innovators in smaller countries will likely have to seek international markets sooner, but this may still be a viable path to success, given that the majority of Shantha's sales occur abroad and that it faces intense competition in the local Indian market that has lowered profit margins.

## Conclusions

Shantha's founder, Dr. Varaprasad, emphasizes the importance of Southern innovation [54]:

*My strong claim is in developing countries these initiatives are necessary. Absolutely necessary. And if there was no such initiative, the Indian populace would have remained not using the vaccine, and consumption would have remained at 180,000 doses. Today it is possible. The government has not done that - we have created awareness. We have conducted mass vaccination camps, and we are giving it at 23 cents which made all people buy it from private doctors. 100 million doses are being consumed. Awareness is there, and the children are protected*.

While initial focus on generics and contract research, as well as reduced patent protection for drugs, has allowed the Indian biotech industry to build expertise and capacity, without incentives and focus on innovation the industry risks falling into the trap of focusing on low risk and profitable drugs rather than important health challenges. As of early 2008, of the 424 home-grown Indian biopharma companies, only 57 (<15%) held US patents [55]. Among biotech firms, this study reported a total of 19 US patents filed from 2001 to 2010. Among these only 2 (11%) were characterized as 'product,' with 9 (47%) being process patents, and seven (37%) both 'product and process' patents and only one (5%), a design patent [55]. With the Indian government having adopted the WTO-TRIPS agreement that emphasizes product patents over process patents, Indian firms will be forced to innovate as they may be unable to afford the royalties to Western products while keeping their prices low [56].

It may become significantly more difficult for new Indian biotechs to emulate Shantha with the higher barriers to innovation for market entry, and existence of a large critical mass of competitors. In 2007, over 15 companies were found to be involved in the marketing of 50 brands for 15 different vaccines in the Rs 3053 crores ($USD 745 million) vaccine market [56]. The cost advantage that India has enjoyed is also diminishing. Home-grown innovative engines like Shantha remain critical; the Global Alliance for Vaccination Initiative (GAVI) continues to resist requests by countries with generic industries for supporting the transfer of patented vaccine technology [13,57] Varaprasad therefore believes that the Indian biotechnology industry cannot afford to continue along the road of generics, or merely serve as an outsourcing shop for Western biotechs.

However, these fears concerning the inability of Indian biotechs to innovate and/or maintain domestic access may have been overstated as studies by GAVI following the implementation of TRIPS in 2006 revealed that all five major Indian vaccine manufacturers had novel vaccine projects for local markets [53]. Moreover, governments maintain the option to use provisions of the Doha Declaration on TRIPS, as well as protections within the TRIPS agreement itself to maintain access to new priority vaccines [58]. Technology transfer is continuing to occur through initiatives such as the Meningitis Vaccine Project, which oversaw the transfer of polysaccharide conjugate technology to local manufacturers, and the Developing Country Vaccine Manufacturers' Network (DCVMN) [59].

GAVI financing has also ensured low pricing by guaranteeing markets for suppliers - when it first began there was only one Haemophilus-influenzae type b (Hib)- containing vaccine available, while there are now four available with three manufactured in emerging markets such as India [53]. And even if prices of vaccines increased due to the need to absorb the increasing cost of innovation, studies show that vaccine introduction by governments often proceeds independently of moderate price differences, and rather depends on national prioritization based on disease burden, competing priorities, and ability to demonstrate meaningful health impact [59]. Moreover, over 95% of drugs that are sold in India are already off-patent, so even if product patents were to eventually raise prices of biologics, the impact would be minimal [56]. These results suggest that the changing intellectual property environment is unlikely to impede the ability for Indian vaccine manufacturers to innovate, or limit access to vaccines by governments.

The globalization of healthcare R&D activities has made it possible for some parts of the developing world to begin to innovatively solve their own health problems. The case of Shantha Biotechnics shows that a billion dollar biotech can be built not only *in *the developing world, but *for *the developing world. More critically, it may be an early sign of the shift of global healthcare R&D away from rich countries to emerging markets. A recent study found that not only do vaccine producers in emerging markets account for over 60% of traditional childhood vaccine doses globally, they now also account for over 20% of innovative products such as combination vaccines - and this latter fraction appears to be growing [53]. Indeed, increasing interest in emerging markets from multinationals, orphan drug-like legislation and innovation platforms reveal that both global health and global wealth might be pursued in parallel [60].

Shantha's affordable high-quality vaccines have already reached hundreds of millions of children globally. The open question that Shantha and Varaprasad have always struggled with is balancing the need for affordable solutions for domestic health needs with focusing on what will increase firm valuation. Governments in the developing world similarly struggle in finding the right balance to reward long-term domestic health innovation, while promoting cheaper solutions for the domestic health gap. The hope is that firm value will align with improving drug access and local health outcomes. Finding a happy medium will be challenging for healthcare innovators in the developing world, but Shantha has shown that it can be done.

## Competing interests

PAS has received consulting funds from Merck Frosst Canada and is on the scientific advisory board of the Bioveda II fund in China.

## Authors' contributions

JC, HM and PAS contributed to the concept and design of this study. HM and JH participated in site visits and data collection. JC, HM, KM and PAS analyzed the findings, and participated in manuscript development. All authors have read and approved the final manuscript.
